# In silico saturation mutagenesis and docking screening for the analysis of protein-ligand interaction: the Endothelial Protein C Receptor case study

**DOI:** 10.1186/1471-2105-10-S12-S3

**Published:** 2009-10-15

**Authors:** Federica Chiappori, Pasqualina D'Ursi, Ivan Merelli, Luciano Milanesi, Ermanna Rovida

**Affiliations:** 1 Institute for Biomedical Technologies – National Research Council (ITB-CNR), via Fratelli Cervi 93, 20090 Segrate (MI), Italy; 2 Consorzio Interuniversitario Lombardo per l'Elaborazione Automatica (CILEA), via Raffaello Sanzio 4, 20090 Segrate (MI), Italy

## Abstract

**Background:**

The design of mutants in protein functional regions, such as the ligand binding sites, is a powerful approach to recognize the determinants of specific protein activities in cellular pathways. For an exhaustive analysis of selected positions of protein structure large scale mutagenesis techniques are often employed, with laborious and time consuming experimental set-up. 'In silico' mutagenesis and screening simulation represents a valid alternative to laboratory methods to drive the 'in vivo' testing toward more focused objectives.

**Results:**

We present here a high performance computational procedure for large-scale mutant modelling and subsequent evaluation of the effect on ligand binding affinity. The mutagenesis was performed with a 'saturation' approach, where all 20 natural amino acids were tested in positions involved in ligand binding sites. Each modelled mutant was subjected to molecular docking simulation and stability evaluation. The simulated protein-ligand complexes were screened for their impairment of binding ability based on change of calculated Ki compared to the wild-type.

An example of application to the Endothelial Protein C Receptor residues involved in lipid binding is reported.

**Conclusion:**

The computational pipeline presented in this work is a useful tool for the design of structurally stable mutants with altered affinity for ligand binding, considerably reducing the number of mutants to be experimentally tested. The saturation mutagenesis procedure does not require previous knowledge of functional role of the residues involved and allows extensive exploration of all possible substitutions and their pairwise combinations. Mutants are screened by docking simulation and stability evaluation followed by a rationally driven selection of those presenting the required characteristics. The method can be employed in molecular recognition studies and as a preliminary approach to select models for experimental testing.

## Background

Structure-based site-directed mutagenesis is a widely used approach to elucidate and modify specific aspects of protein function and to investigate the properties of protein-ligand interactions. Prediction of mutants with desirable properties is often obtained by rational design of a few specific position in the region of interest. This approach requires an in-depth knowledge of physicochemical, structural and functional properties of the protein, which may not be exhaustively provided by X-ray crystallography data. A different strategy is to apply the so-called scanning mutagenesis based on the systematic replacement of all residues involved in a specific function. Several examples of alanine scanning mutagenesis experiments [1] are reported in literature that illustrate its relevance for the study of molecular determinants of ligand binding [2] and protein function [3]. Even more informative is the use of the saturation mutagenesis, where all 20 natural amino acids are tested in place of the wild type residue, in or near the functional site. 'In vitro' scanning saturation mutagenesis has been applied to several case studies and has proved to be useful to modulate protein properties such as substrate enzyme specificity [4] or to identify key residues for catalytic mechanisms [5].

A particular aspect that is amenable to investigation with random mutagenesis is the study of protein-ligand interaction. Several examples are reported in literature that show how this approach has contributed to the clarification of ligand interaction in G protein coupled receptors [6,7], in nuclear receptors [8,9] and in EphA3/ephrin-A5 [10], to cite a few examples.

However, the large number of mutants that are obtained from the systematic replacement of all the residues of a functional site makes the experimental procedure laborious and time consuming. This is especially true when dealing with large functional sites, because it requires the implementation of specific high-throughput methods for mutant screening or selection [11]. 'In silico' simulation of mutagenesis and screening may represent a possible way around this problem. By exploiting the capabilities of high performance computers, it is possible to design and test a large numbers of variants with altered properties in order to restrict the number of models that are worth testing experimentally. Computational protein design methods have been employed in protein engineering to increase enzymes efficiency [12] or antibiotic resistance: for example Hayes et al. [13] applied a protein optimization strategy to increase the resistance of bacteria toward the antibiotic cefotaxime by optimizing TEM-1 β-lactamase. Moreover, computational design tools have been developed and have proved to be successful in the design of proteins with enhanced stability and specificity, and in engineering novel protein functions [14].

We present here a high performance semi-automated computational procedure suited to the study of key residues of protein-ligand interactions, which is able to dissect the contribution of different residues to ligand binding. The procedure performs a saturation mutagenesis of all residues involved in ligand binding, and the subsequent evaluation of the effect of amino acid substitutions on ligand affinity by docking simulation and on protein structure stability. An example of application to Endothelial Protein C Receptor is reported.

## Methods

### Pipeline implementation

A semi automated pipeline was designed to manage mutant modelling, docking simulations and data analysis procedures by integrating public software through a number of in-house developed Perl scripts.

#### Mutant modelling

'In silico' side chain replacement and modelling were carried out with the routine 'mutate_model' of MODELLER 9v3 [15]. 'Mutate_model' introduces a single point mutation in a user-specified residue and optimizes the mutant side chain conformation by conjugated gradient and by molecular dynamics simulation.

#### Docking

AutoDock4.0 [16,17] was used for docking simulation handling both ligand and mutated side chain as flexible. AutoDockTools (ADT) () facility supported the protein and ligand set up for docking. The automatic procedure, based on a Perl script, employs the following ADT routines:

- 'prepare_receptor4' to add polar hydrogens, assign Kollman charges and convert the protein PDB file in pdbqt (the input file for AutoDock4);

- 'prepare_flexdocking' and 'prepare_flexreceptor' are applied to protein and ligand to obtain the input files for flexible docking simulation;

- 'prepare_gpf' and 'prepare_dpf' generate the parameters file for AutoGrid (gpf) and for AutoDock (dpf), respectively.

Docking simulations were performed with Lamarkian Genetic Algorithm which is reported as the most efficient and reliable method of AutoDock [16].

#### Stability evaluation

The 'stability' routine of FoldX algorithm [18] was applied to evaluate the total energy of wild type structure and mutated models by the estimation of the interactions that contribute to the protein stability.

### Computational resource

Docking simulations were performed on a shared Linux cluster of 280 Opteron AMD cores 275 at 2,2 GHz, provided by the Eurotech Group (Amaro, UD, Italy), dedicated to bioinformatics applications. The system is composed of 10 chassis of 6 diskless blades, each equipped with an Infiniband 4× network card and 8 GB of RAM. The cluster nodes are accessed through the OpenPBS queuing system.

### Case study

#### Protein structure and preparation

The crystal structure of the Endothelial protein C receptor (PDB: 1LQV), in a complex with phosphatidylethanolamine (PTY), and the Gla domain of protein C solved by X-ray crystallography at 1.6 Å [19] was retrieved from the Protein Data Bank [20]. Chain A with the corresponding ligand (PTY) molecule was used for this study. The Gla domain, crystallization water, Ca ions, N-acetyl-D-glucosamine (NAG) and 2-(acelytamino)-2-deoxi-A-D-glucopyranose (NDG) were removed from PDB file. Polar hydrogens were added at 7.4 pH, using InsightII (Accelrys, San Diego).

#### Docking simulation

The ADT graphics interface was employed for manual preparation of the ligand to assign the flexibility, add polar hydrogens and load Gasteiger charges. 22 rotatable bonds were assigned to the PTY ligand.

A grid box of dimensions 76 Å × 60 Å × 92 Å was constructed around the binding site, based on the co-crystallized ligand. Ligand flexible docking simulations were performed with 100 runs and 2500000 energy evaluations per run, while the other parameters were set to the default.

## Results

### Pipeline description

**Figure 1:**
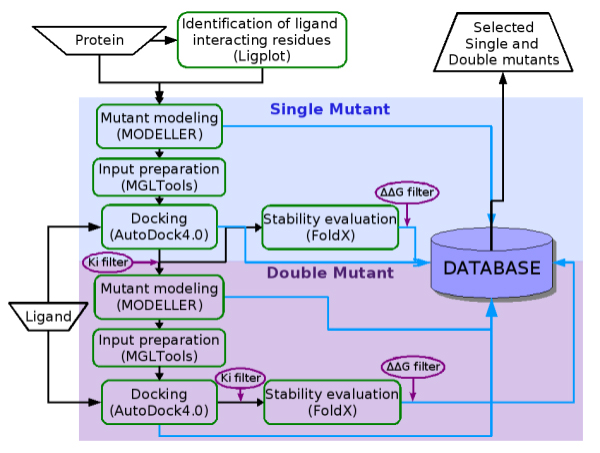
**Schematic diagram of the pipeline**. Blue panel represents the first stage of the pipeline devoted to single mutants design. Violet panel is the second stage devoted to double mutants. Steps within the panels are automatically executed. "Protein" and "Ligand" are the pipeline input files and contain the corresponding atomic coordinates. "Selected single and double mutants" are the pipeline outputs. Steps involving public software are in green boxes. Ki (inhibition constant) and ΔΔG (free energy difference) filters are in purple circles. Black arrows connect different steps of the pipeline, while blue arrows identify the intermediate outputs collected in the database.

The complete scheme of the computational procedure used in this work is depicted in Figure 1. A preliminary step is performed using LIGPLOT [21] to identify the residues involved in ligand interactions. The pipeline has a modular structure to improve its adaptability to different datasets. The backbone of the pipeline is a MySQL database where the results of each computational step are stored (blue arrows in Figure 1). The pipeline is structured in two main stages devoted to the modelling and analysis of single and double mutants respectively (blue and violet boxes).

The first stage begins by single mutant modelling starting from the receptor atomic coordinates and the list of residues identified in the preliminary step. This first step makes use of the MODELLER routine 'mutate_model' that performs the side chain substitution and refinement. A Perl script is used to iterate the substitution of each residue with all the amino acids to achieve the saturation mutagenesis. For each mutant, a PDB file is obtained and stored in the database.

In the following step, the pipeline prepares the input files for AutoGrid and AutoDock4.0 according to user specified parameters: PDB files are converted to PDBQT format, adding polar hydrogens and assigning Kollman charges. Substituted residues are set for flexible docking. The ligand is manually prepared as illustrated in the Methods section and given as input to the pipeline. In the next step, AutoDock simulations are submitted to be run in parallel on the Linux cluster. Docking results are parsed to extract, for each simulation, the most representative conformation. This corresponds to the best energy conformation included in an AutoDock cluster of at least 5 elements and showing a binding energy within one Kcal/mol compared to the first ranked solution. This criterion is arbitrarily set in order to obtain a solution from a well represented group of conformations with the best scored results. However, when these conditions are not satisfied (i.e. no populated clusters are present in the top score), the first ranked solution is accepted. For each selected conformation, the corresponding values of binding energy, cluster population, inhibition constant (Ki), root mean square deviation (RMSD) and atomic coordinates are stored in the database. In order to select mutants with a modified affinity for the ligand compared to the wild type, the output data are filtered taking the wild type Ki value as threshold.

Structural stability of selected mutants is evaluated using the FoldX program that computes, for each conformation, an estimated free energy values. The free energy difference between the mutant and the wild type (ΔΔG = ΔG_mutants _- ΔG_wild type_) is automatically calculated and stored in the database.

Pair combinations of single substitutions are analyzed in the second stage of the pipeline (violet box in Figure 1) to investigate the occurrence of synergistic effects in double mutants. Residues chosen for double mutants modelling are obtained from the results of Ki filtering in first stage screening. A Perl script manages the residues combination, removing the redundant pairs. The following pipeline steps, simulations, data analysis, filtering and storage, are performed as in the first stage.

The database is designed to collect and organize all the output data which remain available for further study. Specific queries can be applied in order to compare binding capabilities and stability of all the mutants, as illustrated in the example below.

### Case study

**Figure 2:**
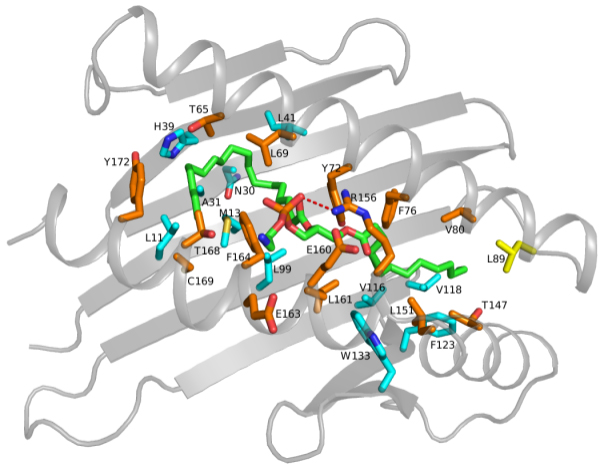
**Cartoon representation of the 3D structure of EPCR-PTY complex**. PTY ligand and EPCR residues involved in interaction, target of saturation mutagenesis, are shown in sticks. Carbon atoms are in green for the ligand, in orange for residues located on α-helices H1, H2 and H3, in cyan for residues of the eight-stranded β-sheet floor and in yellow for the loop residue. In red, the H-bond between Arg156 and the polar group of PTY. Figure is drawn with Pymol (DeLano Scientific, San Carlos, CA, USA).

We used the described strategy to study the ligand binding of the Endothelial Protein C Receptor (EPCR). EPCR is a transmembrane glycoprotein homologous to the major histocompatibility complex (MHC) class 1/CD1 family [22]. EPCR plays a well-characterized role in the coagulation cascade being the receptor of the anticoagulant factor Protein C (PC) and participating to its activation mechanism. Being involved in protein C pathway, EPCR also takes part in the regulation of inflammation and apoptosis in endothelial cells [23]. Similarly to CD1 molecules, it binds a phospholipid ligand in a deep hydrophobic groove composed of an eight-stranded β-sheet floor and three almost parallel α-helices that lie above the floor and form a crevice where the lipid is located (Figure 2) [19]. The specific role of the lipid for the protein structure and function has not yet been clarified. Amino acid variants located in the lipid binding region of EPCR may provide useful information about the role of this interaction in protein function. However, mutations that impair or suppress the lipid binding have not been reported so far. In this work we predicted mutants that have reduced binding affinity for the ligand, while maintaining structural stability.

The structure of human EPCR, co-crystallized with PTY, exhibits a wide pattern of interactions that extend the length of binding groove. PTY makes contacts with 27 residues: four are located on α-helix H1, two on H2 and nine on H3, one on the loop between H1 and β-strand 5, and eleven residues on the strands of β-sheet floor (Figure 2). The binding is mostly dominated by hydrophobic interactions with the hydrocarbon chain of the ligand; a single H-bond is established between the polar group of phospholipid and ARG 156.

**Figure 3:**
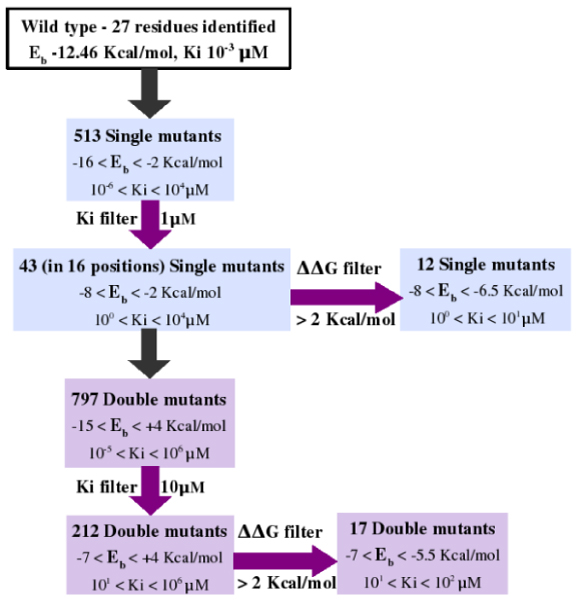
**Summary of results for the case study**. Eb (binding energy) and Ki (inhibition constant) values obtained by docking simulation of the wild type EPCR with PTY are reported. For each step of the analysis (blue and violet boxes), the number of mutants and the corresponding Eb and Ki ranges are represented. Ki and ΔΔG (free energy difference) filters used for mutant screening are also indicated. The analysis starts with the mutagenesis of 27 residues that yields a total of 1310 (513 single and 797 double) mutants. At the end of the screening, the number is reduced to 12 single and 17 double mutants.

Employing the pipeline described above, we have carried out an 'in silico' saturation mutagenesis of the EPCR residues involved in lipid binding. Each modelled mutant was subjected to molecular docking simulation and stability evaluation. The protein-ligand complexes obtained by docking simulations were screened by their inhibition constant (Ki) values in order to estimate the impairment of ligand binding ability induced by the mutation. The overall procedure and a summary of the results are depicted in Figure 3. The 27 interacting residues, identified using LIGPLOT, were saturated with the 19 remaining amino acids, producing 513 mutant models. Then, wild type and mutants were analysed by docking simulations. The wild type re-docking correctly reproduced the X-ray ligand conformation with a RMSD of 1.2 Å, a binding energy of -12.46 Kcal/mol and a Ki value of 1 nM. Positions and substitutions critical for binding affinity were identified with a Ki-based filter. Referring to the wild type Ki value, we used a threshold of 1 μM (i.e. 3 order of magnitude higher than wild type) to discriminate between mutants with probable impaired binding ability. The pipeline selected 43 mutants with Ki value higher than the threshold, substituted in 16 out of the 27 starting positions. Pair combinations of the 43 substitutions were generated and 797 double mutants were modelled. They were analyzed by the same docking protocol of the single mutants and filtered with a Ki threshold of 10 μM. This value was set an order of magnitude higher than for single mutants, with the purpose of selecting those combinations with a binding ability worse than the single ones. With this filtering criterion, 212 double mutants were rescued. The large reduction in the number of double mutants (from 797 to 212), suggested that most residue combinations did not cooperate to increase the Ki value compared to the single substitutions.

The structural stability of single and double variants that passed the corresponding Ki thresholds was evaluated using the FoldX tool, directly integrated in the pipeline. Mutants ensuing a ΔΔG value higher than 2 kcal/mol were considered structurally unstable and removed from the selection [24]. After the screening, the pipeline retrieved 12 single and 17 double mutants with a ΔΔG < 2 Kcal/mol. Binding energy and Ki values intervals are reported in Figure 3. The stability filter was passed only by mutants with a Ki value within 10 and 100 μM for single and double, respectively. This value was an order of magnitude higher than the respective Ki thresholds. Mutants with high Ki values, although potential candidates for binding affinity studies, were rejected by the ΔΔG filter and thus considered unstable.

**Figure 4:**
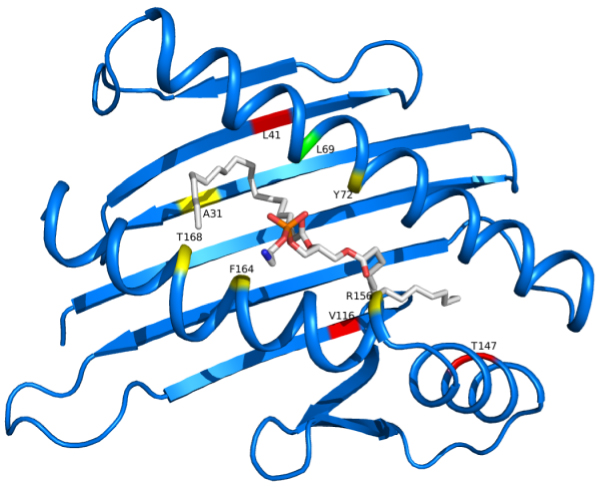
**Key positions replaced in mutants with impaired ligand binding**. Wild type EPCR residues are represented as colored spots corresponding to the Cα position. L69 (in green) was found substituted only in single mutants. Residues substituted in both single and double mutants are in yellow, while those observed in double mutants only are in red. PTY ligand is represented in sticks. Figure is drawn with Pymol (DeLano Scientific, San Carlos, CA, USA).

At the end of this analysis, 6 key positions for the ligand binding in EPCR were identified (Figure 4): position 69 and 72 are localized in the H1 α-helix, positions 156, 164 and 168 are localized in H3 α-helix and one, the position 31 on the eight-stranded β-sheet floor. The most represented substitutions of these key positions involved hydrophobic residues, such as W, in positions 31, 69 and 156, and L, in positions 31 and 164.

**Figure 5:**
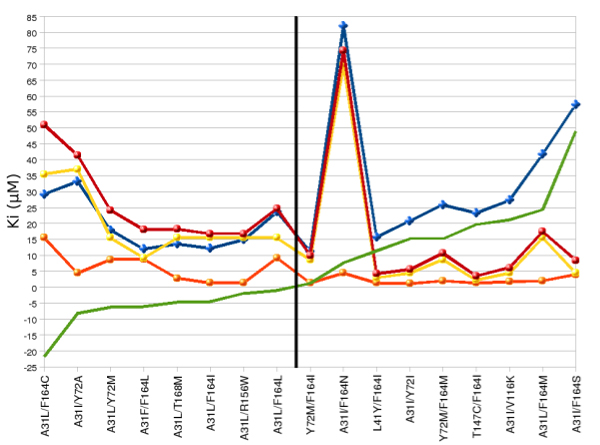
**Synergistic effect in selected double mutants**. The effect was evaluated as the difference (Ki_d_, green line) between the Ki value of the double mutant and the Ki value of the sum of the two corresponding single mutants. Ki_1+2_: Ki values of double mutants (blue diamonds); Ki_1_, Ki_2_: Ki values of the two single mutants (yellow and orange dots, respectively); Ki_1_+Ki_2_: sum of the Ki values of the two corresponding single mutants (red dots).

Pair combinations in double mutants were evaluated in terms of synergistic effect where the Ki value of double mutant was larger than the sum of the corresponding single ones. To estimate the synergistic effect, we applied the following equation:

(1)

where Ki_1 _and Ki_2 _represent the Ki value of the first and the second single mutant, and Ki_1+2 _the Ki value of double mutant. Ki_d _is the difference between the effect of the double mutation (Ki_1+2_) and the sum of the two corresponding single mutations (Ki_1_+Ki_2_). Results are shown in Figure 5. We found that 8 of the 17 double mutants displayed a partial additive effect as the Ki value was included between the more damaging mutation and the sum of the two corresponding single mutants (Ki_1 _< Ki_1+2 _< Ki_1_+Ki_2_) (on the left of the black vertical line, in Figure 5) [25]. The remaining 9 double mutants had Ki values higher than the sum of the two corresponding single mutants (Ki_1+2_> Ki_1_+Ki_2_), to which we assigned a synergistic effect compared to the single mutation (on the right of the black vertical line, in Figure 5). We observed that the highest Ki values were associated with double mutants resulting from replacement of A31 with either I or L and F164 with a small polar/apolar side chain. Between them, the highest synergistic effect was shown by A31I/F164S mutant (Ki_d _= 49), while the largest effect in terms of binding energy (-5.58 Kcal/mol) and Ki (82 μM) value was associated to A31I/F164N mutant.

In conclusion, using the described approach, we have selected a limited number of single and double mutants of EPCR for which we predict a reduced binding ability for the PTY ligand and a structural stability comparable to the wild type. The set of 12 selected single mutants and the 9 double mutants presenting synergistic properties can be proposed as rationally constructed candidates for experimental testing in cellular models.

### Pipeline performance

Due to the computational load needed to accomplish all the simulations, a parallel infrastructure was employed (see Methods). In this test case, the simulation of the single and double mutant took on average 12 and 15 hours, respectively. The whole challenge consisted of 513 docking experiments for the single mutants plus the wild type simulation and 797 for the double mutants. Depending on the resource availability, it was possible to employ 80 CPUs on average. Therefore, in a period of about ten days we collected all the results covering about 2 years of computer activity.

## Conclusion

We present a high performance semi-automated computational pipeline that integrates modelling and docking simulation algorithms for large scale mutant design. Additional analysis tools such as structure stability evaluation are also included. The pipeline is organized into two main blocks for dealing with single and double mutants that are interconnected so that selected results of first analysis are used for the second part of the study. The procedure is presently devoted to protein-ligand interaction studies, but can be adapted to investigations of protein-protein interaction. The mutagenesis method is based on a saturation approach and does not require previous knowledge of functional or structural role of involved residues: it can therefore be applied to explore new binding features. The implementation of the pipeline on a parallel computer infrastructure permits a greatly reduced computational time and makes it possible to test a very large number of mutants. For example, in the reported text case we have modelled and tested 1310 variants of EPCR with the purpose of identifying single or coupled amino acid mutations that significantly impair the binding of the lipid ligand. The whole procedure reduced the number of selected models to 21, which corresponds to about 2% of the initial dataset. Selected variants provide information about the probable functional and structural effect of the mutated residues and can be proposed as subjects for experimental tests. In addition, the large amount of data stored in the database after each step of computational analysis can be further retrieved for more detailed examination and study. This pipeline can be useful for protein-ligand binding studies to identify key residues for the ligand interaction, to evaluate the effect of the substitutions on different ligands and to design selective mutants.

## Competing interests

The authors declare that they have no competing interests.

## Authors' contributions

FC developed the core of the pipeline, tested all the software for modelling and docking simulations, prepared the biological dataset, analyzed the output results and drafted the manuscript. PD conceived and designed the study and critically revised the results. IM contributed to developing the pipeline, designing the database and enabling the parallel computation of the docking experiments on the cluster infrastructure. LM supervised the project and made available the computational resources. ER coordinated the study and critically revised the results and manuscript. All authors read and approved the final manuscript.
